# On Inflammation of the Choroid Coat
*Glasgow Journal, No. IX.


**Published:** 1830-07-01

**Authors:** 


### XXXVII.

### On Inflammation of the Choroid Coat.*

Our attention having been particularly drawn to a paper bearing the above title, from the pen of our former confrère, and a very able surgeon, Mr. Mackenzie, we were induced to con it over with care. The perusal justifying the encomia pronounced on it by our correspondent, we shall introduce it to the notice of a far wider circle of readers, than the original respectable journal in which it appeared can possibly obtain for it.

The situation and characters of the choroid coat, says Mr. Mackenzie, supply sufficient reasons for the inflammation which attacks its texture, having hitherto failed to attract attention or having bid defiance to precise elucidation. It is generally acknowledged that iritis is occasionally attended by inflammation of the choroid, but as the arteries of the parts are distinct in course and distribution, the idea of a separate choroiditis and iritis is, à priori, rendered probable.

"For some time, the separate existence of choroiditis was with me rather a matter of speculation, and a conclusion from analogy, than a fact ascertained by observation. I am now convinced, however, that the choroid is sometimes the seat, almost quite independently, of inflammation; that in certain cases of ophthalmia, it is the focus of the disease, and that the neighbouring parts may be as little affected when that is the case, as the sclerotica is in iritis, or the iris in sclerotitis. That it is of importance to distinguish the disease which I am now about to describe, will appear very evident, when we consider its dangerous nature. Its symptoms, as we shall immediately see, are very different from those of any other ophthalmia; and although ultimately the whole eye may be involved by inflammation commencing in the choroid, yet choroiditis, in the early stage, exists without any signs of disease in the iris, and without any other effects upon the sclerotica and retina, than those which must necessarily arise from the pressure of an inflamed and swoln membrane, placed in contiguity with other membranes, more or less susceptible of suffering from that pressure. I consider choroiditis, therefore, as completely a primary and distinct disease."

Symptoms. 1. *Discoloration of the White of the Eye.* From the pressure of the inflamed choroid the exterior tunics become extenuated, so that the dark choroid shewing through the sclerotica gives to it a blue or purplish hue. This is a very remarkable and early symptom, whilst the degree of discoloration varies with the severity and duration of the attack, and is best observed by comparing the healthy with the morbid eye.

2. *Tumour.* When the discoloration has existed for a time the affected part protrudes, commonly on one side only of the eye-ball near the cornea, as if the corpus ciliare was the seat of the disease, and most frequently at its superior or temporal side. The tumour may enlarge to the size of half a filbert or more, and is then generally of a deep blue, with varicose vessels running over it, a state to which the name of sclerotic staphyloma has been given. Several such tumours may surround the cornea.

"The front of the eye, however, is not the only seat of choroid staphyloma, as it might be called with more propriety than sclerotic, considering the actual origin of the protrusion. Scarpa, tells us that he had never met with any tumour or elevation of the sclerotica on its anterior surface, resembling a staphyloma; but that he had twice happened to meet, in the dead body, with staphyloma of the posterior hemisphere of the sclerotica. The first time was

---

* Glasgow Journal, No. IX.

in the eye of a woman of forty years of age. The eye was of an oval figure, and upon the whole, more voluminous than the sound eye on the other side. On the posterior hemisphere of the diseased eye, and to the external or temporal side of the entrance of the optic nerve, the sclerotica was elevated in the form of an oblong tumour, like a small nut. As the cornea was sound and pellucid, and the humours still preserved their natural transparency, on looking through the pupil, there appeared towards the bottom of the eye, an unusual brightness, produced by the light penetrating that part of the sclerotica, which had become thin and transparent where it was occupied by the staphyloma. When the eye was opened, the vitreous humour was found entirely disorganized, and converted into limpid water, and the chrystalline lens somewhat yellowish, but not opaque. When the posterior hemisphere of the eye was immersed in spirit of wine, with a few drops of nitrous acid added to it, in order to give the retina consistence and opacity, it was distinctly perceived that there was a deficiency of the nervous expansion of the retina within the cavity of the staphyloma; that the choroid was very thin at this part, deprived of its natural colour, and of its usual vascular network; and that the sclerotica, particularly at the apex of the staphyloma, was so thin as scarcely to equal the thickness of writing paper. The woman from whom this eye was taken, had lost the faculty of seeing on that side some years before, during an obstinate ophthalmia, attended with most severe, and almost habitual pains in the head.

Scarpa had an opportunity of making similar observations on an eye met with accidentally by Dr. Monteggia of Milan. It was taken from a woman, thirty-five years of age, was of an oval figure, and longer than its fellow. The staphyloma was situated exactly as in the former instance. The vitreous humour was dissolved; the chrystalline capsule was distended by a thin whitish fluid; the lens yellowish, and less than natural; the retina deficient within the staphyloma; the choroid and sclerotica, forming the tumour, thinned, so as to transmit the light. Nothing positive could be ascertained regarding this woman's sight."

3. *Effusion between the Choroid and Retina.* There can be no doubt that the vessels of the choroid are greatly enlarged in this disease, indeed our author remembers having seen a preparation in the hands of Professor Beer, in which the varices were as large as small peas. Frequently also there is an effusion of watery fluid, and sometimes coagulable lymph, between the choroid and retina; the former Mr. M. has often had occasion to evacuate with a needle. If this be not done, it accumulates to such a degree as to press the retina before it, and having occasioned the absorption of the vitreous humour, it gathers the retina into a cord, which looks, through the pupil, like a deep-seated cataract, or the advancing tumour in medullary fungus. The latter is still more closely counterfeited by the effusion of coagulable lymph between the choroid and retina. In one case such an effusion was occasioned by a penetrating wound of the eye, and the lymph lay in white masses, which remained unchanged so long as Mr. Mackenzie could watch the case. In another instance, however, the lymph has assumed a red colour, and pressed forward almost into contact with the cornea. The same appearances have been noticed by Mr. Travers.

4. *Redness.* The arteries of the distended sclerotica are much enlarged in choroiditis, and not unfrequently we observe a patch of redness near the edge of the cornea, fed by one or more of these dilated vessels. Sometimes the redness is confined to the upper part of the eye-ball; there is seldom any of the conjunctiva, but either it is sclerotic, or consists in an enlargement of the visible ar eries derived from the recti muscles.

5. *Displacement of the Pupil.* "The iris is not affected with inflammation in choroiditis; but the pupil, in almost every case which I have witnessed, has undergone a remarkable change of place. The iris is always narrowed towards the portion of the choroid which is affected, and in many instances, the pupil is observed to have moved so much out of its natural situation, as to be almost directly behind the edge of the cornea. Upwards, and upwards and outwards, are the directions in which the pupil is most frequently observed to become displaced. It occasionally continues small and moveable, in other cases it is immoveable, but not dilated; in very severe cases it is greatly enlarged, the iris having entirely disappeared at that part of its circumference towards which the diplacement of the pupil has happened.

"The remarkable displacement of the pupil which attends choroiditis is owing probably to some affection of one or more of the ciliary or iridal nerves, which running forward between the sclerotica and choroid, pass through the annulus gangliformis, and ultimately reach the iris. This symptom has been remarked by Beer as an attendant on syphilitic iritis. That it is not a constant attendant is well known. I have seen it in other varieties of iritis. It has never been attributed to any affection of the choroid, nor has any explanation of its cause been offered.

"The pupil does not return to its place, even although the choroiditis is subdued."

6. *Opacity of the Cornea.* This is a frequent, though not necessary attendant on choroiditis, and generally it is the edge of the cornea nearest the portion of affected choroid that becomes opaque, whilst the rest remains perfectly clear. In other cases there are pretty extensive but irregular spots of whiteness, rather the effect of interrupted nutrition than of inflammation. In some severe and long-continued cases of choroiditis the cornea becomes almost entirely opaque, and even dilated and staphylomatous, when this alone may destroy the patient's vision.

7. *Exophthalmos and Exophthalmia.* "In consequence of choroiditis, the eye may enlarge, and even protrude from the orbit to a very considerable degree, without much inflammation of the sclerotica and conjunctiva, these tunics being merely thinned by the pressure of the distended choroid. After a time, however, the eye in this state of exophthalmos, is apt to suffer from external inflammation, in consequence of being but imperfectly protected by the lids, or it may be, in consequence of cold or mechanical injury. When the inflammation, thus excited, runs to a great height, the conjunctiva becomes chemosed, puriform fluid is deposited behind the cornea, or between its lamellæ, the eye bursts, continues to swell and protrude still more, assumes a fungous appearance, bleeds profusely, and being productive of great pain and deformity, evidently requires to be extirpated."

8. *Intolerance of light and epiphora*, are generally considerable.

9. *Pain.* This varies much in different individuals. It is moderate when there is no protrusion; it becomes severe and sometimes furious, when the sclerotica is much pressed and distended, especially when this takes place suddenly, and is attended with considerable increase of redness. Hemicrania is also present, affecting principally the top of the head, the high part of the temple, and the cheek. It is not strictly circumorbital, nor is it strikingly nocturnal.

10. "*Vision* is variously affected in cases of choroiditis. In some, the very first symptom complained of, is dimness of sight. Hemiopia, all objects to one or other side of a perpendicular line, or above or below a horizontal line, appearing dim, all objects appearing confusedly, and as if double, even when viewed with one eye, are symptoms which not unfrequently distress the patient long before any redness or blueness of the eye is visible. If the disease goes on, we sometimes find that total blindness ensues, even when the choroid appears but partially affected; while in other cases the whole choroid is evidently affected, the whole eyeball enlarged and discoloured, and yet a considerable degree of vision is retained."

CONSTITUTIONAL SYMPTOMS. The subjects of this disease are adults, and those of strumous habit are most subject to it. Various degrees of febrile excitement attend it; in the early stage the pulse is not affected, but after the patient has suffered much, an extremely cachectic state is apt to follow. The digestive organs are frequently much deranged from the very first, and continue to be so throughout the disease.

CAUSES. Mr. Mackenzie attributes the commencement of the disease to want of exercise; derangement of the digestive organs; over use of the eyes in reading, sewing, miniature-painting, and other minute works; exposure to too much heat and light, the glare of hot fires, vicissitudes of temperature; and finally, blows on the eye and penetrating wounds.

PROGNOSIS. Recovery is always slow. If the disease has gone to any considerable length, it is scarcely ever completely removed. The vestiges of it are in general permanent, even after it has been completely checked in its progress. In many cases, we may reckon ourselves fortunate, if we arrest this disease. Yet it sometimes happens that the cure proceeds to a degree beyond our expectation. I lately attended a gentleman who, many years before, had almost entirely lost the sight of the left eye from this disease. The right was now attacked. Both pupils were greatly displaced; the visible arteries of the right eye were much dilated, and the sclerotica at different places considerably extenuated; the left eye was enlarged, of a pretty deep blue colour, and a great part of the cornea opaque. By blood-letting, counter-irritation, and other remedies, the disease was arrested in the left eye, and very unexpectedly the right eye recovered to such a degree, that he was again able to read with it an ordinary type.

TREATMENT. 1. *Blood-letting.* Profuse and repeated blood-letting does more good in the early stage of choroiditis, than all other remedies put together. Yet we might perhaps not be tempted to bleed sufficiently at this period of the disease, from the circumstance that in many instances, there are no external signs of intense inflammation, and the patient does not suffer any acute pain. The practitioner, therefore, who is not acquainted with the nature and symptoms of this ophthalmia, might be apt to trifle away time in the application of a few leeches, when he should be opening the temporal artery, and removing a large quantity of blood. I have known the blueness and evident distention of the sclerotica, which, notwithstanding leeching and other remedies, had continued unabated for many weeks, disappear suddenly and completely, after the loss of twenty or thirty ounces of blood from the temple. Bleeding from the jugular vein, or from the arm, is also highly useful. Twenty-four or more leeches round the eye, every second day, I have seen attended by the best effects. In chronic cases, we must not neglect the frequent and liberal application of leeches.

2. *Purgatives* are of essential service. The disordered state of the biliary and other digestive organs, indicates the use of calomel as a cholagogue, followed by salts and senna, or some other brisk purgative. Such remedies are to be repeated frequently, during the course of the treatment.

3. *Mercury.* We are naturally led to advise mercury in choroiditis, from observing its happy effects in iritis. But on the whole, I must confess, that in the former disease, I have not witnessed any remarkable benefit, either from making the mouth sore, or from small doses long continued. I have used this medicine both in friction to the head, and in various forms internally; but it has appeared inert so far as the choroiditis is concerned. Still, I have hitherto continued to prescribe mercury in this disease, because the cases which I have treated are too few to enable me to decide completely on this point, and because this medicine is found to do good in all other chronic inflammations of the eye.

4. *Turpentine* I have lately tried in one or two cases, but am unable as yet to come to any conclusion regarding its effects.

5. *Iodine.* In one case only have I

fully tried this powerful sorbefacient, and I am happy to say, with an amount of good effects altogether unlooked for. An eye which I had many times punctured, and had fairly made up my mind to extirpate, has shrunk considerably under the use of the tincture of iodine, while the sclerotica has assumed much more of its natural whiteness.

6. *Tonics.* After due depletion, I have seen much benefit accrue from the precipitated carbonate of iron, and the sulphate of quina. They may be given separately, or together.

7. *Counter-irritation* is decidedly useful. A tartar emetic eruption between the shoulders is perhaps the most effectual.

8. *Paracentesis oculi.* Puncturing the sclerotica and choroid, so as to evacuate the aqueous fluid collected between the latter tunic and the retina, is a remedy of much importance in the treatment of this disease. It is not to be tried in the acute stage, at least I have not dared to try it except in the chronic stage, and when there was an evident tendency to staphyloma scleroticæ. The operation is performed with a broad cataract-needle, which is to be thrust, not in the direction of the lens, which it might readily wound and render opaque, but towards the centre of the vitreous humour. The instrument need not penetrate deeper than the eighth part of an inch. A little blood is usually discharged from the divided portion of the choroid, mixed with aqueous fluid of a slightly glutinous consistence. The operation gives great relief to the feeling of distention or pressure in the eye, and to the attendant headach. It may be repeated every eight days, or at longer intervals, according to the state of the eye.*

9. *Extirpation of the Eye* becomes necessary, when the organ is much protruded, and at the same time destroyed by inflammation, or when organized lymphatic exudation within the eye, (a symptom over which paracentesis has no control,) goes to such a length that the tunics are kept constantly in a state of painful distention. The eye having fallen into either of these conditions, the general health of the patient is apt to suffer so much from continued irritation, want of sleep, inability to take exercise, and loss of appetite for food, that there can be no doubt of the propriety of removing a part which has become entirely unfit for its office, and remains only as a source of disturbance to the constitution."

This concludes Mr. Mackenzie's very able and instructive memoir on choroiditis. To say that we have analyzed it would be untrue, for it is written with so much brevity that small room was given us for further abbreviation. We strongly recommend its attentive perusal to our readers, and perhaps the most solid and most flattering compliment we can pay the worthy author, is the assurance that the transplantation of his paper to our pages, will introduce it into company and circles, with which it could not otherwise have formed so much as even a bowing acquaintance. An introduction in this case is all that can be wanted, because to be courted and admired, it only requires to be known.

### XXXVIII.

### LITHOTRITY.

* See a case of Staphyloma Scleroticæ successfully treated, by repeatedly tapping the Eye; by Richard Martland, M.D., in the Edinburgh Medical and Surgical Journal. Vol. xxiii. p. 59. Edin. 1825.

